# Pharmacist-Led Audits for Older Adults With Cancer Yield Significant Interventions

**DOI:** 10.1093/geroni/igaa057.672

**Published:** 2020-12-16

**Authors:** Hilary Erdeljac, Stephanie Yager, Megan McClain, Sarah Wall, Carolyn Presley, Edmund Folefac, Ashley Rosko

**Affiliations:** 1 The Ohio State University, Columbus, Ohio, United States; 2 James Cancer Hospital and Solove Research Institute at The Ohio State University Wexner Medical Center, Columbus, Ohio, United States; 3 The James Cancer Hospital, The Ohio State University, Columbus, Ohio, United States

## Abstract

Older adults with cancer have comorbidities that require medical management and confounders of chemotherapy and supportive medications exacerbate polypharmacy. A multidisciplinary team model was created to address these needs within the Cancer Aging and Resiliency (CARE) clinic. To reconcile medications for accuracy, compliance, side effects, and effectiveness, a pharmacist-led audit includes identification of potential therapeutic duplications, drug-drug interactions, or potential medication inappropriateness identified using Beers criteria. A pharmacist led review of patient’s prescriptions can identify drug therapy problems (DTP) and result in safer medication management. METHODS: A retrospective review of pharmacy specific interventions was conducted using CARE Clinic patient data from February 2016 to October 2019 evaluating data from n=259 patients. RESULTS: A preliminary analysis of n=137 patients who had received medication reconciliation were included. The mean number of medications per patient was 13.1 ± 5.7 and 457 DTP were identified leading to 523 medication related interventions. There was an average of 3.3 DTP per patient. The most common types of DTP included medication reconciliation (n=137, 30.0%), potentially inappropriate medication (PIM) (n=74, 16.2%), administration/technique (n=35, 7.7%), and drug-drug interaction (n=28, 6.1%). The most frequent types of interventions involved education to the patient (n=166, 31.7%), medication reconciliation (n=137, 26.2%), medication discontinuation (n=84, 16.1%), patient to discuss further with physician (n=39, 7.5%), and medication initiated (n=35, 6.7%). Updated results involving approximately 259 patients will be presented. CONCLUSION: Comprehensive medication review within a multidisciplinary setting for the management of older adults with cancer can reduce polypharmacy and inappropriate medication use.

